# The effects of CA IX catalysis products within tumor microenvironment

**DOI:** 10.1186/1478-811X-11-81

**Published:** 2013-10-29

**Authors:** Alice Santi, Anna Caselli, Paolo Paoli, Denise Corti, Guido Camici, Giuseppe Pieraccini, Maria Letizia Taddei, Sergio Serni, Paola Chiarugi, Paolo Cirri

**Affiliations:** 1 Dipartimento di Scienze Biomediche Sperimentali e Cliniche, Università degli Studi di Firenze, Viale Morgagni 50, 50134 Firenze, Italy; 2 CISM Centro di servizi di Spettrometria di Massa, Università degli Studi di Firenze, Viale G. Pieraccini 6, 50139 Firenze, Italy; 3 Dipartimento di Medicina Sperimentale e Clinica, Università degli Studi di Firenze, Largo Brambilla 3, 50134 Firenze, Italy

**Keywords:** Tumor microenvironment, CA IX, Extracellular pH, Coculture, Bicarbonate, Cancer associated fibroblasts, Tumor metabolism

## Abstract

Solid tumors are composed of both cancer cells and various types of accessory cells, mainly fibroblasts, that collectively compose the so called tumor-microenvironment. Cancer-associated fibroblasts have been described to actively participate in cancer progression by establishing a cytokine-mediated as well as metabolic crosstalk with cancer cells. In the present paper we show that activated human fibroblasts are able to boost tumor cells proliferation and that this effect is greatly dependent on stromal carbonic anhydrase IX (CA IX) activity. In fact fibroblasts show a strong upregulation of CA IX expression upon activation by cancer cells, while CA IX products, protons and bicarbonate, exert differential effects on cancer cells proliferation. While acidification of extracellular pH, a typical condition of rapidly growing solid tumors, is detrimental for tumor cells proliferation, bicarbonate, through its organication, supplies cancer cells with intermediates useful to sustain their high proliferation rate. Here we propose a new kind of fibroblasts/tumor cells crosstalk within tumor microenvironment, mediated by stromal CA IX products, aimed to favor cancer cells growth, opening new perspectives on CA IX role in tumor microenvironment.

## Background

Solid tumors are not only a collection of highly heterogeneous cancer cells but a very complex tissue composed by both cellular and non-cellular components, commonly defined as the tumor microenvironment (TM). The major cellular components include fibroblasts, endothelial and immune cells, that collectively produce the variety of molecules representing the non-cellular components of the tumor stroma, i.e. the extracellular matrix (ECM) proteins, proteases, cytokines and growth factors. The main stromal cellular component of TM is an “activated” form of fibroblasts called Cancer Associated Fibroblasts (CAFs) [1]. The trans-differentiation of CAFs is mediated by numerous growth factors and cytokines secreted by cancer cells such as Tumour Growth Factor-1 (TGF-β1) [2], PDGF-α/β [3] and interleukin (IL)-6 [4]. Many ways in which CAFs could support cancer progression have been described: a) by secreting growth factors (HGF, EGF, bFGF) and cytokines (SDF-1 and IL-6) leading to the infiltration of immune cells, which in turn promote *de novo* angiogenesis helping metastatic spread [5]; b) by producing an altered ECM that was reported to influence the proliferation, survival and migration of cancer cells [6]; c) by actively remodelling the ECM through the secretion of matrix metalloproteinases (MMP-9, MMP-13 and MMP-14) that are mandatory for tumor progression [7]; d) by engaging a metabolic interplay with cancer cells in which glycolytic fibroblasts produce and secrete lactic acid that is metabolized by oxygenated tumor cells [8].

The tumor microenvironment is also markedly different from that of normal tissues also regarding important extracellular parameters such as extracellular pH (pHe) and oxygen tension (pO_2_). In fact, due to the defective neovascularization of the growing tumor [9], perfusion tends to be heterogeneous and, overall, inadequate [10]. In addition the high metabolic rate of tumor cells leads to a massive production of acidic metabolites (CO_2_, lactic acid) that, in order to maintain the correct intracellular pH (pHi), have to be secreted in the extracellular medium. Hence, the combined effect of defective vascularization and the high metabolic activity of cancer cells leads to the consequence that tumor microenvironment is both relatively acidic and hypoxic compared to normal tissue [11,12]. The stringent control on cellular pHi, mandatory for cells survival [13], is mediated by different types of pHi regulating proteins such as: Na/H exchanger-1, anion exchangers, Na/bicarbonate cotransporters, monocarboxylate transporters and carbonic anhydrases (CAs) [14] .

In particular, one member of the CAs family, CA IX, which is induced by hypoxia, is overexpressed in many tumors, and it is associated with cancer progression and response to therapy [15]. Carbonic anhydrases (CAs, EC 4.2.1.1) are widespread metalloenzymes in higher vertebrates, including humans [16]. Sixteen CA isoforms have been characterized to date in mammals, which differ in their subcellular localization, catalytic activity, and susceptibility to different classes of inhibitors. There are cytosolic isozymes (CA I, CA II, CA III, CAX, CAXI, CAXII, CA VII and CA XIII), membrane bound ones (CA IV, CA IX, CA XII, CA XIV and CA XV), mitochondrial (CA Va and CA Vb) and secreted (CA VI) isoforms. Most CAs are very efficient catalysts for the reversible hydration of carbon dioxide to bicarbonate and protons (CO_2_ + H_2_O ↔ HCO_3_^-^ + H^+^), a reaction of critical importance in most organisms since the bicarbonate/carbonic acid system is the main buffer in all living cells.

CA IX and CAXII are overexpressed in many solid tumors, and are associated with tumor progression and poor prognosis in various human tumors [17,18]. CA IX, in particular, possesses several peculiarities that fit with tumor physiology: a) CA IX is the most strongly overexpressed gene in response to hypoxia [19], a very common condition in fast growing solid tumors, being under the transcriptional control of HIF1-α; b) CA IX possesses an extracellular catalytic domain acknowledged of a remarkable high catalytic activity [20], for that reason being one of the most important protein involved in tumoral extracellular acidification; c) CA IX shows an optimal catalytic activity at pH 6.49 [21], which is a pH value within the range of that of acidic and hypoxic tumors.

In the present work we have studied tumor cells proliferation in *in vitro* coculture models of tumor microenvironment, in particular evaluating the role of CA IX in this context.

## Results

### Cancer cells proliferation within tumor microenvironment

**Figure 1:**
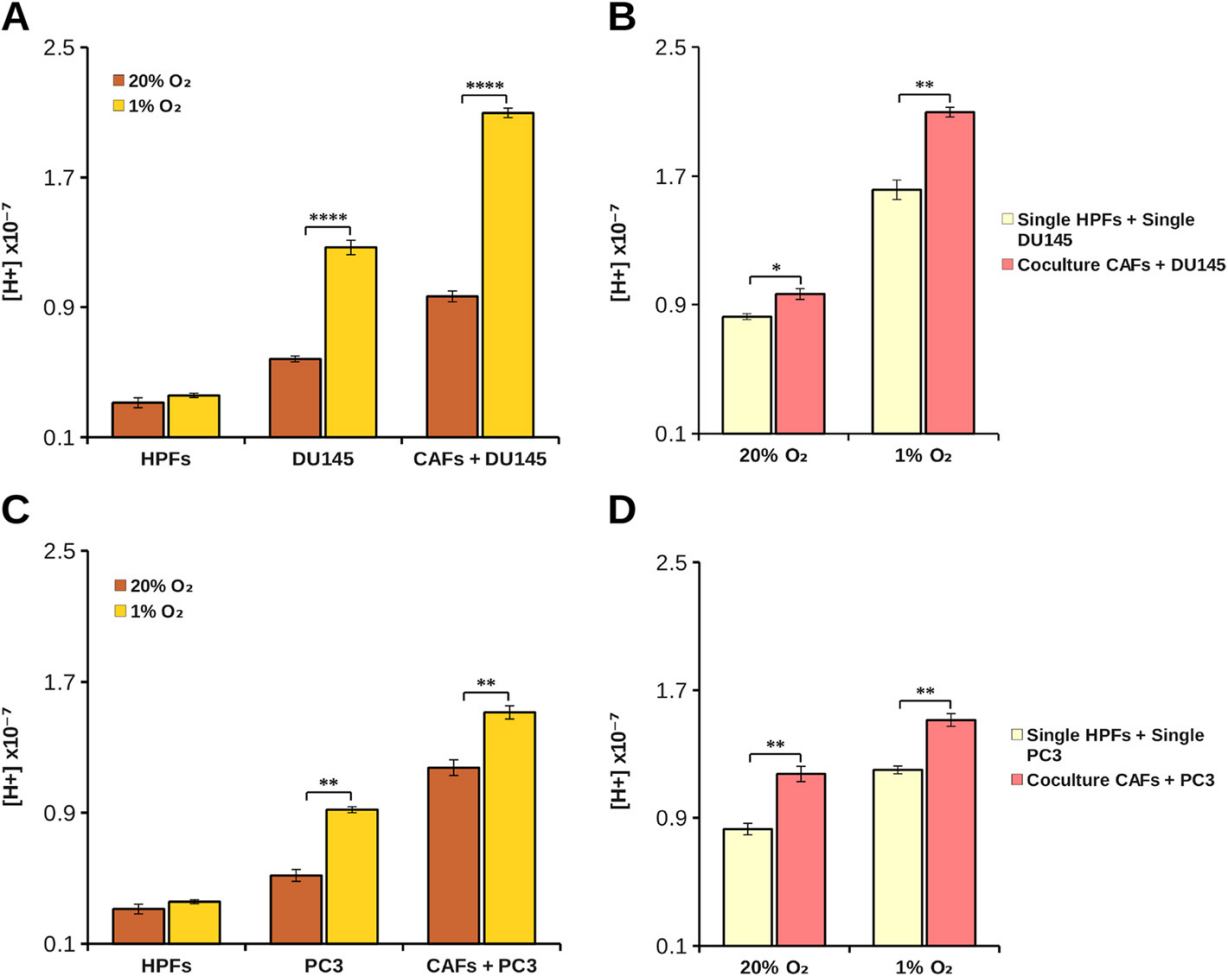
**Effect of hypoxia and coculture on extracellular pH.** Tumor and stromal cells were plated in single culture (2x10^5^ for each cell type) and in coculture (10^5^ DU145 or PC3 and 10^5^ fibroblasts) at 37°C in unbuffered DME medium containing 1 mM bicarbonate in CO_2_ free atmosphere either at 1% O_2_ or 20% O_2_. Results show protons concentration values measured in DU145 single and coculture **(A-B)** and in PC3 single and coculture **(C-D)** after 24 hours calculated as a means ± SD from five independent experiments (*, p < 0.1; **, p < 0.05 and ****, p < 0.005).

**Figure 2:**
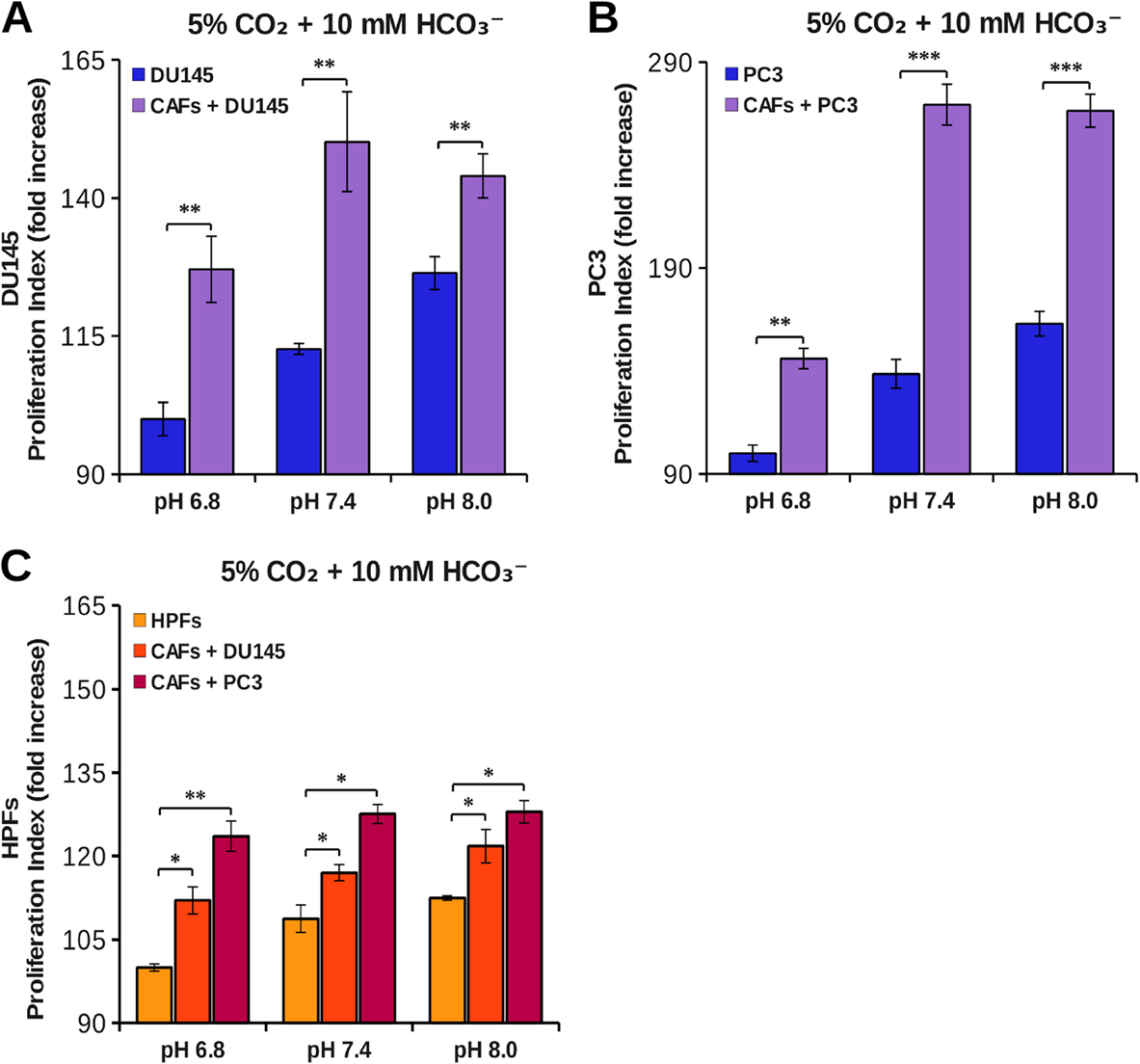
**Effect of acidity on tumor cells growth.** DU145 **(A)**, PC3 **(B)** and HPFs **(C)** were labeled with CFDA-SE and then plated in single culture and in coculture. The cells were grown at various extracellular pH in DME supplemented with 25 mM hepes and 10 mM bicarbonate at 37°C and 5% CO_2_. After 40 hours cells were detached and analyzed by flow cytometry. The data show the fold increase of proliferation index calculated on an average of five experiments (*, p < 0.1; **, p < 0.05 and ***, p < 0.01).

**Figure 3:**
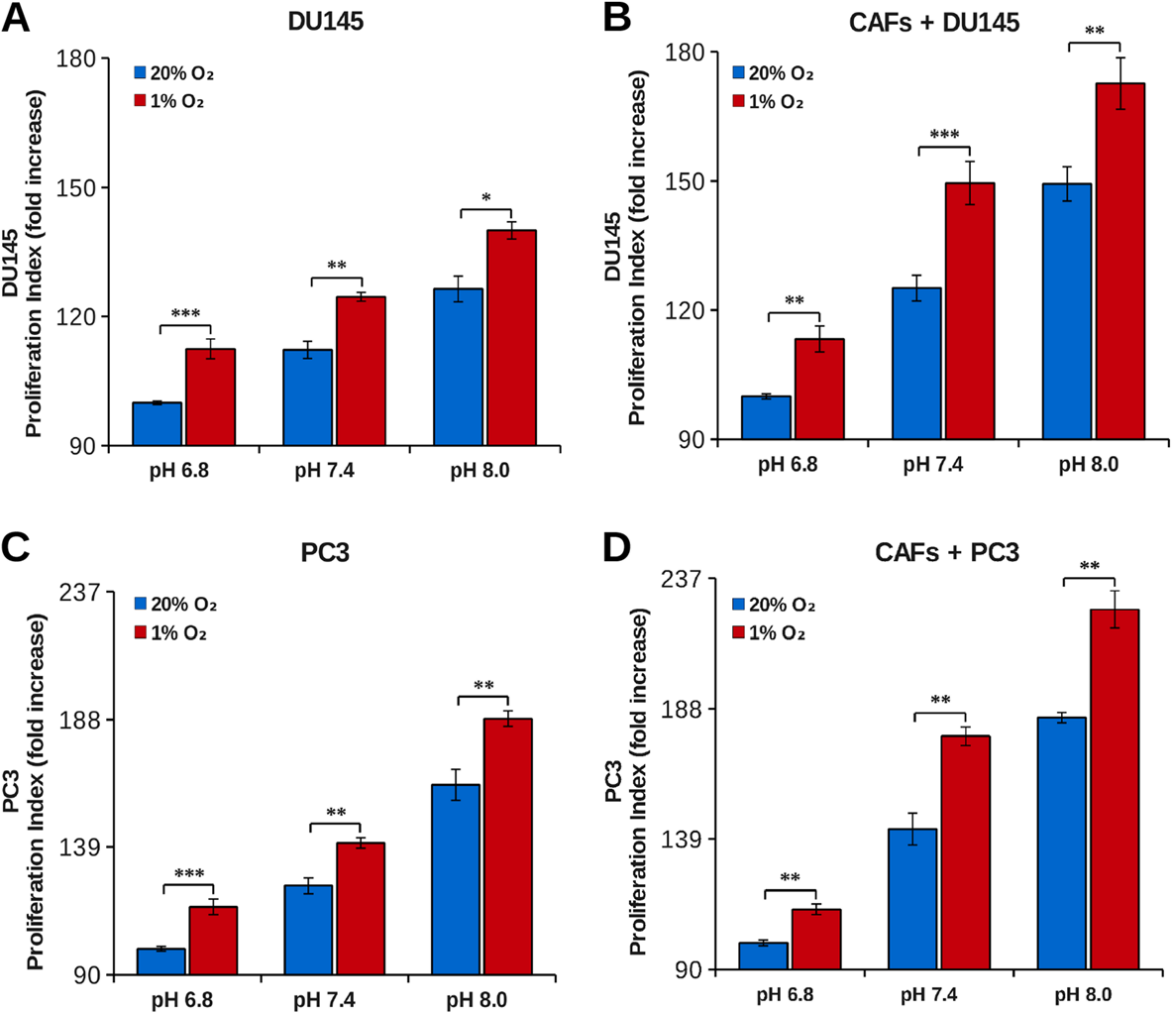
**Effect of acidity on tumor cells growth in hypoxic condition.** DU145 **(A-B)** or PC3 **(C-D)** cells were labeled with CFDA-SE and then plated in single culture and in coculture with fibroblasts in a 1:2 ratio at 1% O_2_ and at 20% O_2_. The cells were grown at various pHe in DME supplemented with 25 mM hepes and 10 mM bicarbonate at 37°C and 5% CO_2_. After 40 hours the cells were detached and analyzed by flow cytometry. The data show the proliferation index calculated on an average of six experiments. (*, p < 0.1; **, p < 0.05 and ***, p < 0.01).

Our aim was to study cancer cells proliferation in the tumor microenvironment context. Our experimental model is based on prostatic tumor cells (PC3 or DU145) and prostatic primary fibroblasts (HPFs) that, following activation by cancer cells, acquire the typical CAFs phenotype [4]. First, we found that tumor cells kept in hypoxia acidify the extracellular medium more than normoxic cells (Figure 1A-C) and that, in coculture, the protons release in the extracellular medium is greater than the sum of single culture cell proton release (Figure 1B-D). This suggests that the interplay between cancer and stromal cells affects important changes in normal and/or in tumor cells metabolism leading to an “extra” acidification of the medium. In order to evaluate the ability of CAFs to contribute to tumor growth at physiological (7.4) as well as acidic (6.8) or basic (8.0) pH, DU145 and PC3 were plated and grown for 40 hours in single or in coculture with CAFs at various pHe, using CFDA-SE cytofluorymetric assay to measure cells proliferation rate of a given population in cocultured conditions (see Methods). We found that single culture cells proliferation rate of both DU145 and PC3 was significantly decreased at acidic pHe (Figure 2A-B), without notable presence of cell death in the pHe range considered (data not shown). In the presence of CAFs, tumor cells proliferation increases at every pHe value compared to the corresponding single culture (Figure 2A-B). However, in coculture, CAFs attenuate the growth inhibition observed in single culture at pH 7.4 compared to pH 8.0, but the negative effect of the lowest pH 6.8 is only partially offset. In addition, the CAFs proliferation rate is significantly greater compared to HPFs one (Figure 2C), although the difference is less extensive respect to the reciprocal effect of fibroblasts on cancer cells (Figure 2A-B), suggesting that the interplay between cancer cells and fibroblasts is mainly devoted to promote tumor cells proliferation. In order to assess the effect of extracellular acidity in environmental conditions more similar to *in vivo* condition, we compared the growth rate of both PC3 and DU145 cells kept at 1% O_2_ at various pHe. Our results show that hypoxic conditions induce a significant increase in tumor cells growth rate compared to tumors kept at 20% O_2_ (Figure 3A-C). Nevertheless, in hypoxia we observed a pH dependence cell growth similar to the normoxic conditions, and the same, strong, pro-proliferative effect exerted by CAFs on cancer cells proliferation (Figure 3B-D).

### Bicarbonate ion contributes to cancer cells proliferation

**Figure 4:**
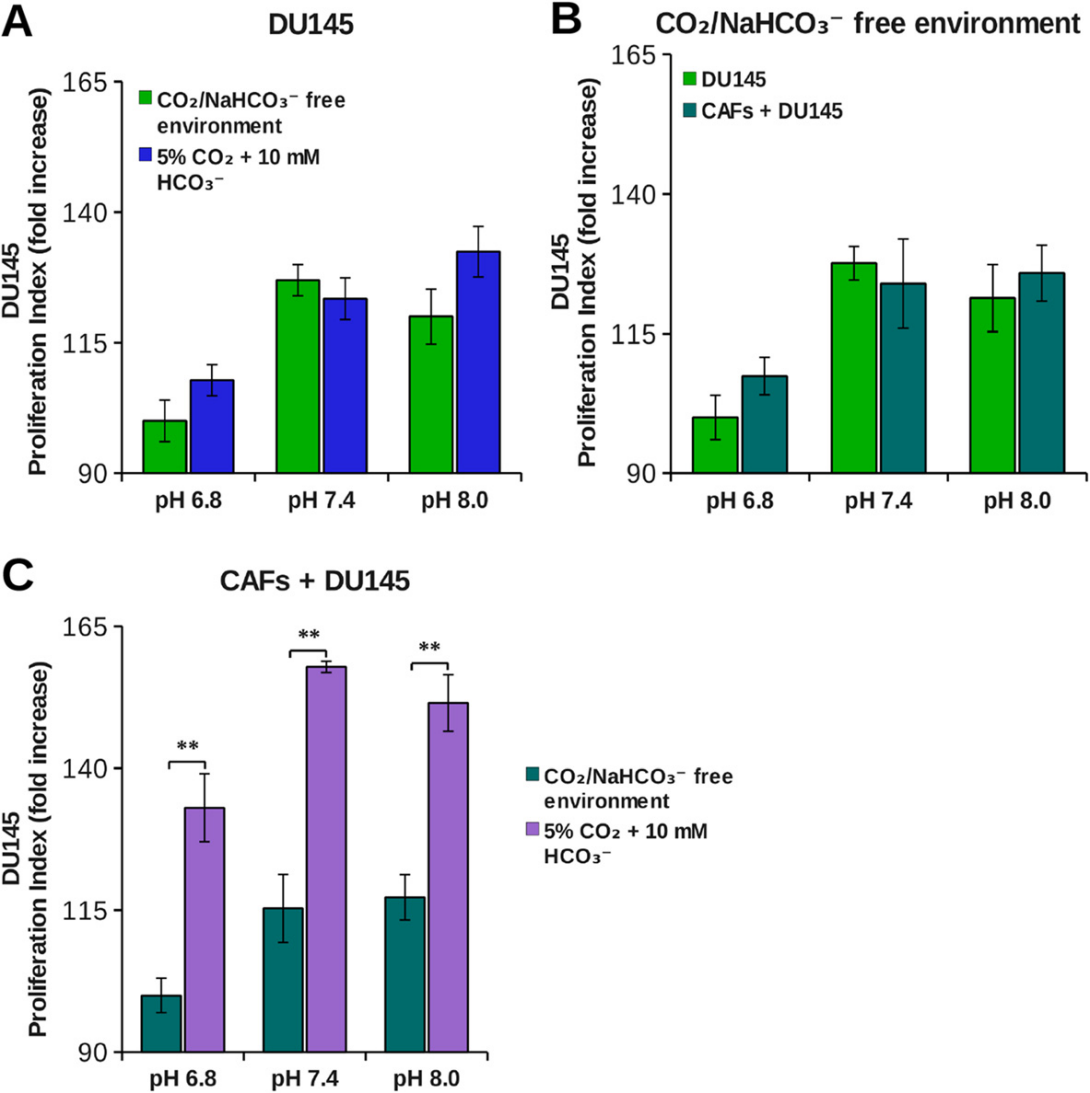
**Effect of bicarbonate on DU145 tumor cells growth.** DU145 were labeled with CFDA-SE and then plated in single culture **(A)**, in coculture with fibroblasts in a 1:2 ratio **(C)** or both **(B)**. The cells were grown at various pHe in DME supplemented either with 25 mM hepes and 10 mM bicarbonate at 37°C and 5% CO_2_ or in DME supplemented only with 25 mM hepes at 37°C in CO_2_/bicarbonate free environment. After 40 hours cells were detached and analyzed by flow cytometry. The data show the proliferation index calculated on an average of four experiments (**, p < 0.05).

**Figure 5:**
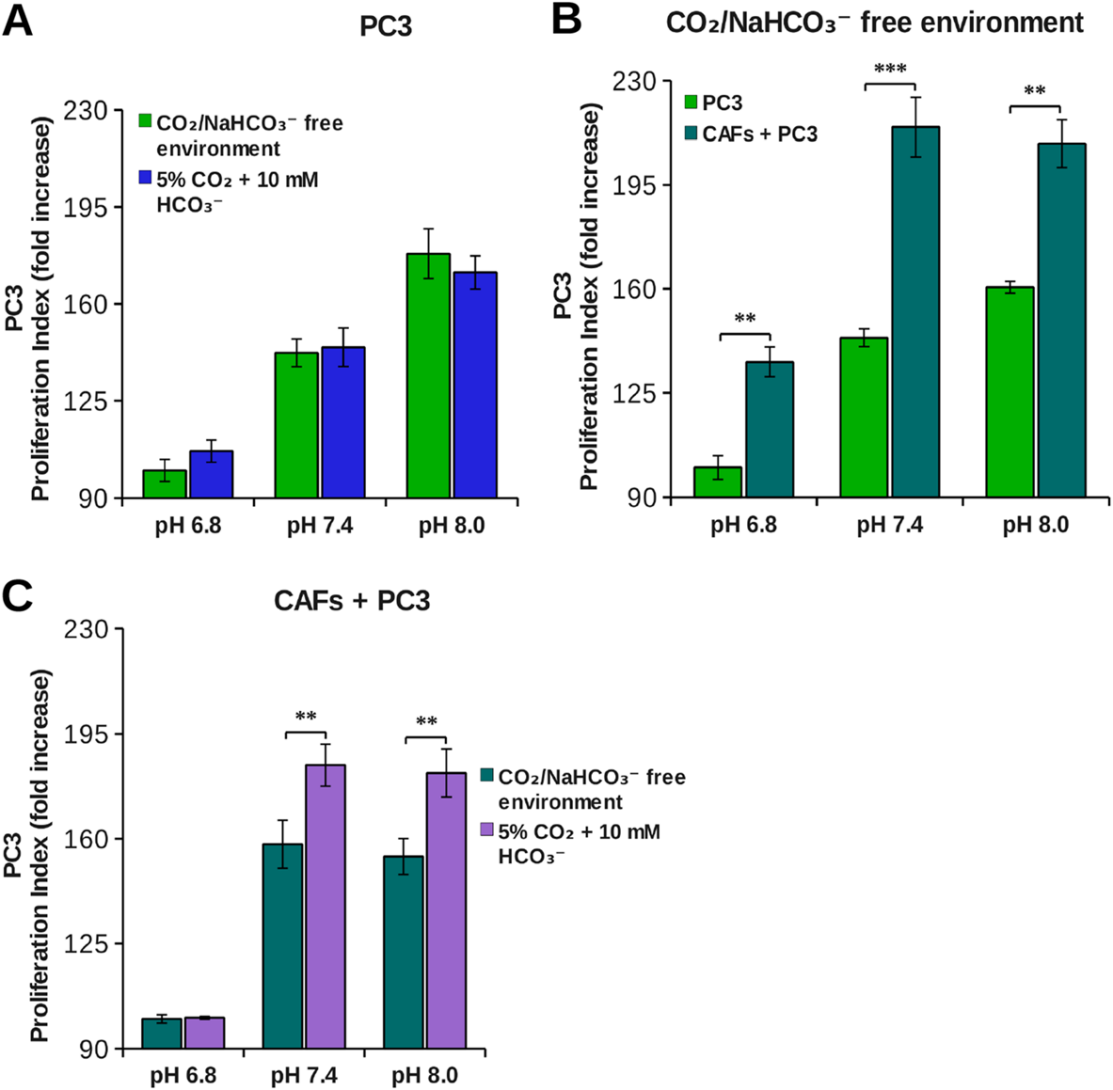
**Effect of bicarbonate on PC3 tumor cells growth.** PC3 were labeled with CFDA-SE and then plated in single culture **(A)**, in coculture with fibroblasts in a 1:2 ratio **(C)** or both **(B)**. The cells were grown at various pHe in DME supplemented either with 25 mM hepes and 10 mM bicarbonate at 37°C and 5% CO_2_ or in DME supplemented only with 25 mM hepes at 37°C in CO_2_/bicarbonate free environment. After 40 hours cells were detached and analyzed by flow cytometry. The data show the proliferation index calculated on an average of four experiments (**, p < 0.05 and ***, p < 0.01).

**Figure 6:**
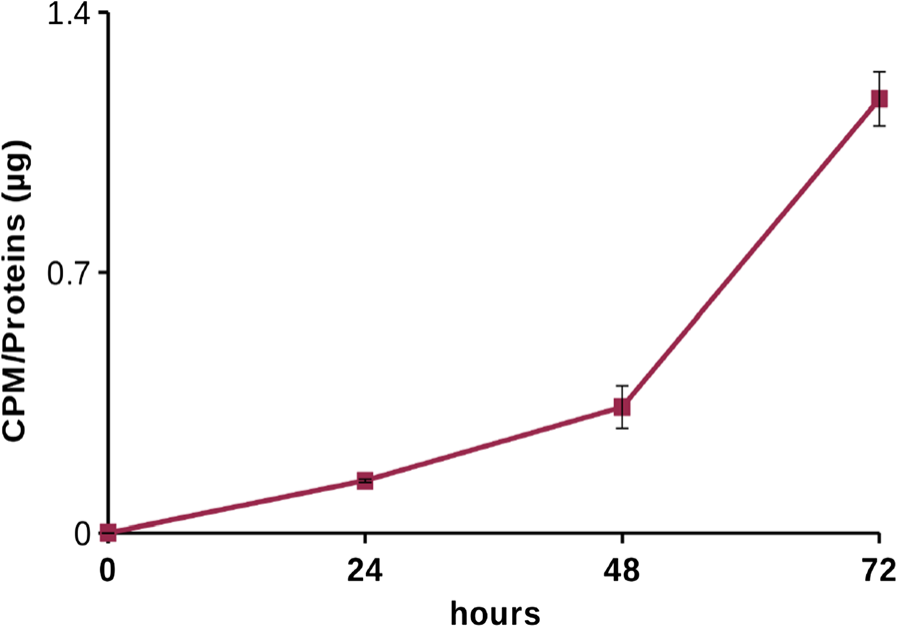
**Bicarbonate organication.** DU145 were cultured in DME supplemented with 25 mM hepes, 10 mM sodium bicarbonate, 20 μM sodium bicarbonate [^14^C] and 10% fetal bovine serum at pH 8.0 for 24, 48 and 72 hours. The data show the CPM (counts *per* minute) value normalized on the protein concentration of each sample, calculated on an average of three experiments.

The bicarbonate buffering system is essential to ensure the homeostasis of both pHi and pHe. The CO_2_ reversible hydration is catalyzed by both intra- and extracellular carbonic anhydrases (CAs). In particular, extracellular CAs are involved in extracellular acidification as well as in bicarbonate production. As shown in Figures 2 and 3, a high concentration of H^+^ ions has negative effects on cancer cells proliferation. Thus, we investigated the possible involvement of the other product of CO_2_ hydration, i.e. bicarbonate, in tumor cells growth. Therefore we compared the growth rate of tumor cells at 5% CO_2_ (which corresponds to CO_2_ pressure present in peripheral tissues) in bicarbonate buffer, with that kept in CO_2_-free environment (i.e. at atmospheric CO_2_ concentration, 0.032% CO_2_). Our results show that in single cultures the proliferation index of tumor cells (DU145 and PC3) is similar in both conditions, therefore bicarbonate does not influence cells growth rate (Figures 4A-5A). In cocultures, instead, we found a different behavior in the two tumor cell lines. For DU145, we did not observe any difference in growth rate between single culture and coculture in CO_2_-free environment (Figure 4B), while CAFs exerted a powerful pro-proliferative effect in the presence of 5% CO_2_/bicarbonate buffer (Figure 4C). In this condition about 90% of the increase in growth rate in the presence of CAFs is due to the presence of bicarbonate for each pHe value considered (Table 1). For PC3 cells, instead, the increase in growth rate due to the presence of CAFs occurs partly also in CO_2_-free environment (Figure 5B), bicarbonate being responsible only for about 30% of the overall growth rate increase in coculture (Figure 5C and Table 1). Hence, whereas CAFs contribution to DU145 proliferation is essentially due to bicarbonate, PC3 cells largely depend on CAFs for their cell growth, but bicarbonate is not the main factor that contributes to this effect. In order to gain information on the use of bicarbonate by cancer cells we performed experiments with ^14^C-bicarbonate that demonstrate the time-dependent organication of bicarbonate (Figure 6). In addition, by using ^13^C-bicarbonate and GC-MS, we found an increased steady state of DU145 intracellular ^13^C-fumarate and ^13^C-malate isotopes when they are grown in coculture with CAFs compared to single DU145 culture (Table 2). This evidence indicates an increase in the bicarbonate organication flux in highly proliferative conditions.

**Table 1:** Relative contribution of bicarbonate to DU145 and PC3 growth rate increase in coculture

	
**Relative contribution of bicarbonate in *DU145 *** **growth rate increase in co-culture (%)**	
	**5% CO**_**2**_ **+ 10 mM HCO**_**3**_^**-**^
**pH 6.8**	82%
**pH 7.4**	100%
**pH 8.0**	89%
**Relative contribution of bicarbonate in *PC3 *** **growth rate increase in co-culture (%)**	
	**5% CO**_**2**_ **+ 10 mM HCO**_**3**_^**-**^
**pH 6.8**	1%
**pH 7.4**	25%
**pH 8.0**	38%

The relative contribution of CO_2_/bicarbonate was calculated taking into account the relative DU145 and PC3 growth rate increase in the presence or in the absence of CO_2_/bicarbonate in the same conditions.

**Table 2:** Isotopic analysis by GC-MS

	^**13**^**C/**^**12**^**C (%)**		
	**DU145**	**DU145 after coculture**	
**Malate**	35.068 ± 0.518	36.341 ± 0.619	*p < 0.05*
**Fumarate**	24.504 ± 0.239	25.258 ± 0.317	*p < 0.05*

Isotopic ^13^C/^12^C ratio of malate and fumarate extracted from DU145 single culture and from DU145 cells separated after being cocultured 40 hours with CAFs. Statistical analysis was performed on the basis of three independent experiments.

### The role of stromal cells CA IX in promoting cancer cells growth

**Figure 7:**
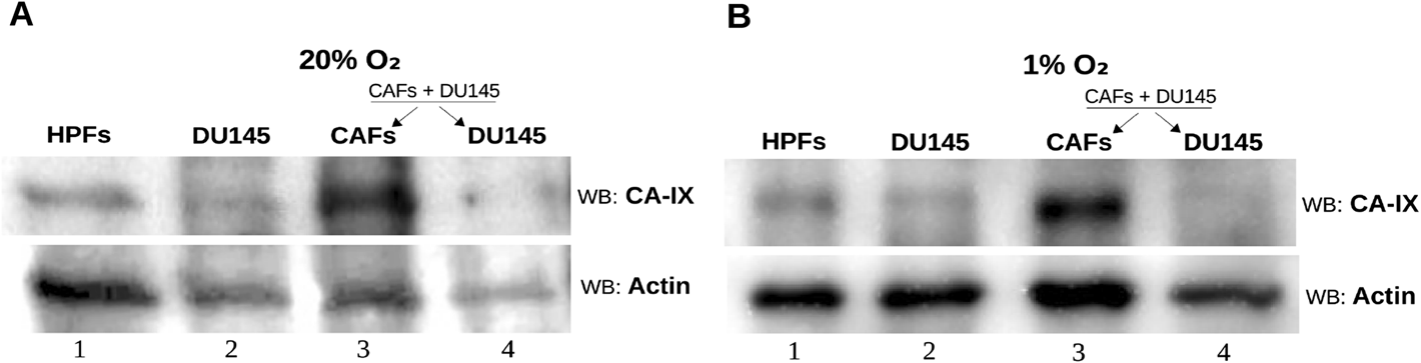
**Analysis of CA IX expression in single culture and in coculture.** DU145 and fibroblasts were plated in single culture and in coculture in a 1:2 ratio for 40 hours at 20% O_2_**(A)** and 1% O_2_**(B)**. After that the coculture were detached and separated by MACS Column Technology. The samples were lysed in SDS-Laemmli Sample Buffer and used for Western Blot analysis. The membranes were treated with anti-CA IX and anti-actin antiboby. Lanes 1 and 2: HPFs and DU145 in single culture. Lanes 3 and 4: CAFs and DU145 after coculture and separation.

**Figure 8:**
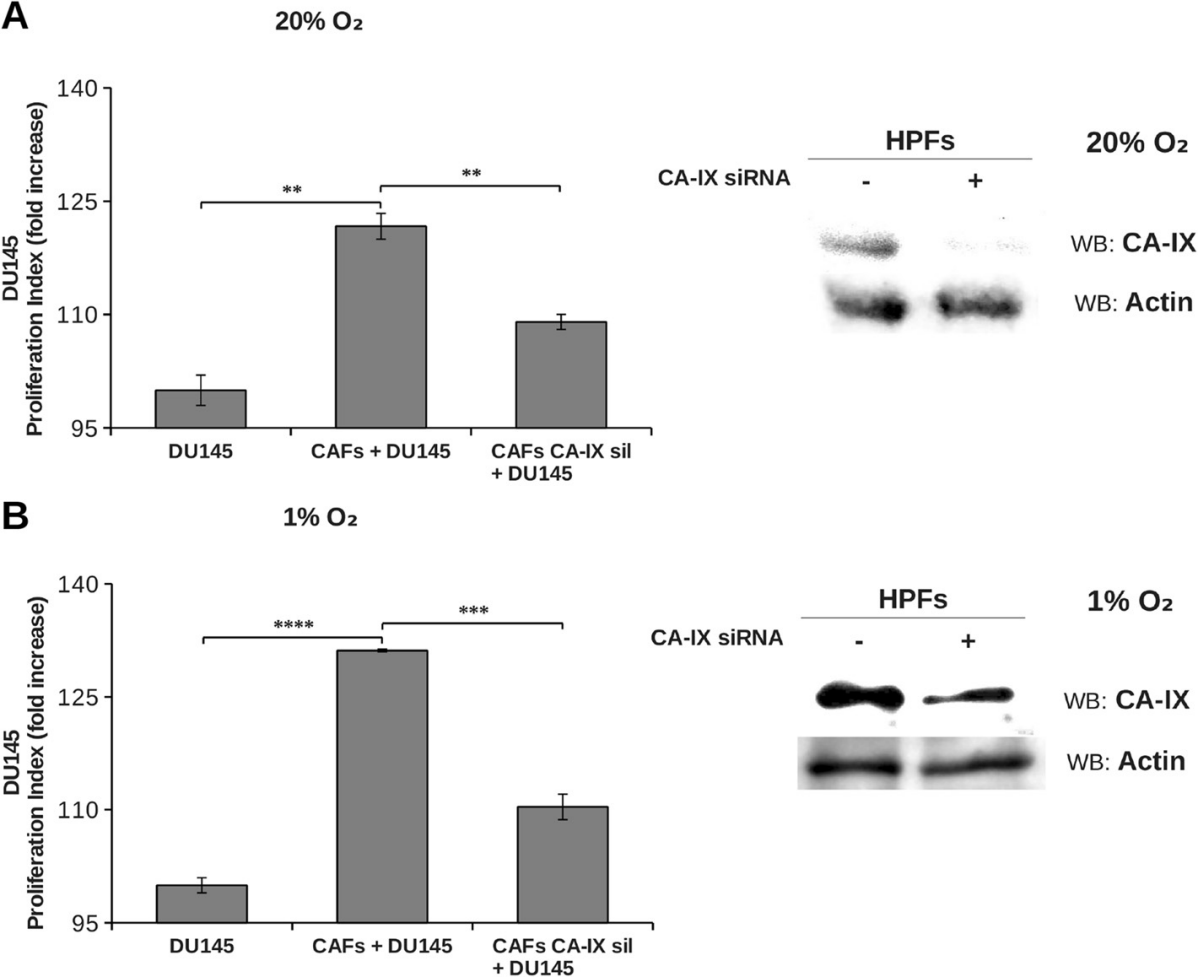
**Effect of fibroblast CA IX downregulation on tumor cells proliferation.** DU145 were labeled with CFDA-SE and then plated in single culture and in coculture with fibroblasts knocked down for CA IX gene or non-silenced, in a 1:2 ratio at 20% O_2_**(A)** and 1% O_2_**(B)**. After 40 hours cells were detached and analyzed by flow cytometry. The data show the proliferation index calculated on an average of four experiments (**, p < 0.05, ***, p < 0.01, ****, p < 0.005). Western blot images show the analysis of CA IX expression in non-silenced or knocked down fibroblasts at 20% O_2_**(A)** and 1% O_2_**(B)**.

**Figure 9:**
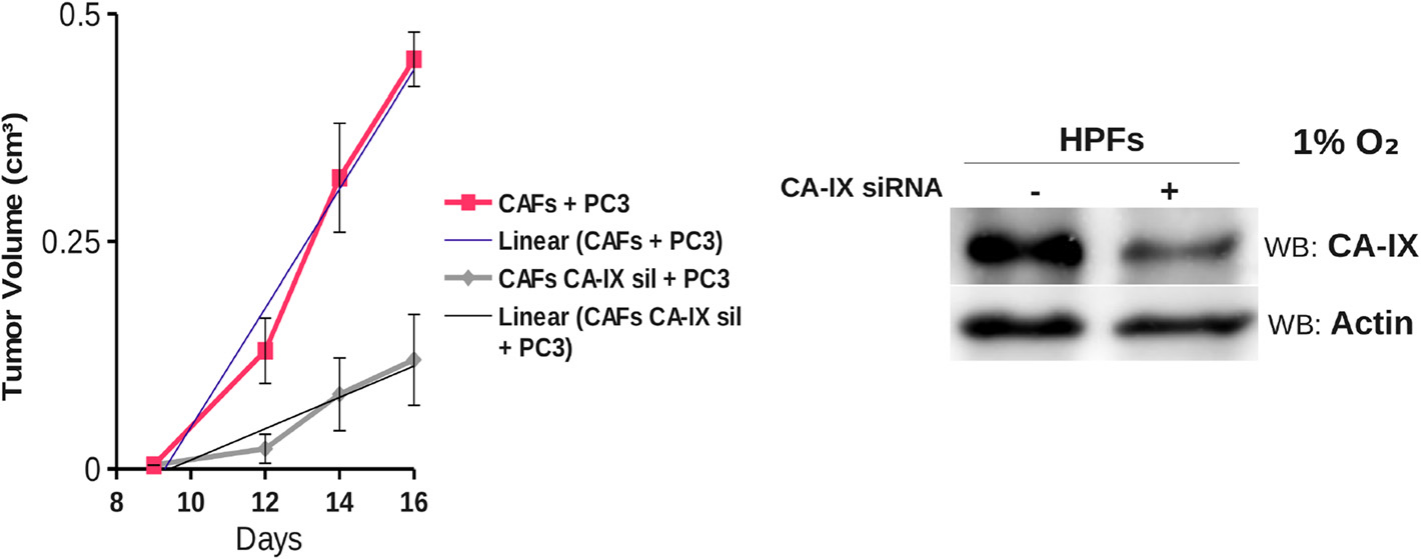
**Silencing of CA IX in fibroblasts decreases tumor growth rate.** Xenograft growth in SCID bg/bg mice of wild-type (control) or CA IX-silenced CAFs injected s.c. with PC3 cells (CAFs:PC3 cells ratio 1:5). CA IX silencing by RNA interference in fibroblasts is shown (Timepoint 14 days: ***, p < 0.01; timepoint 16 days: ****, p < 0.005). Linear regression equation of 'CAFs + PC3′’: y = 0.08065 (± 0.01035) x – 0.82478 (± 0.14473), R^2^ = 0.9676; linear regression equation of 'CAFs CA IX sil + PC3′: y = 0.023219 (± 0.003469) x – 0.249096 (± 0.051038), R^2^ = 0.9563.

DU145 cells growth was significantly fostered in coculture condition exclusively in presence of bicarbonate/CO_2_ buffering system, hence we determined the expression level of CA IX, the main responsible for extracellular bicarbonate production, in DU145 and in fibroblasts in single culture and after 40 hours of coculture at 1% and 20% O_2_ followed by MACS separation of the two cellular populations. We observed that CA IX expression in single culture is low both at 1% O_2_ and 20% O_2_, while it greatly increases only in CAFs after 40 hours of coculture with DU145 at both oxygen tensions (Figure 7). PC3 were less dependent on bicarbonate for their growth in coculture (Figure 5C and Table 1), accordingly we observed a reduced induction of stromal CA IX expression due to PC3 presence (see Additional file 1) compared to that observed after activation of fibroblasts with DU145 (Figure 7). In order to assess the role of CAFs CA IX in promoting cancer cells growth, we silenced stromal CA IX and measured DU145 growth rate in coculture conditions. Our results (Figure 8) show that CAFs knocked down for CA IX are far less able to support cancer cells proliferation respect to non-silenced counterpart. In fact, the growth rate of DU145 in presence of CAFs knocked down for CA IX decreases both at 1% O_2_ and at 20% O_2_ and gets close to that of the single culture. Finally, to assess the role of stromal CA IX in promoting *in vivo* tumor growth, we co-injected PC3 cells and CAFs (1:5 ratio) in the lateral flanks of SCID-bg/bg mice and we observed that fibroblasts knock-down for CA IX are less able to sustain tumor mass growth compared to control experiment with non-silenced fibroblasts (Figure 9). Linear regression fitting on these data shows that tumor volume growth rate is about three times lower in presence of CAFs defective for CA IX expression compared to wild type ones.

## Discussion

Solid tumors are composed of both highly genetically heterogeneous cancer cells and by numerous kinds of non transformed cells composing the so called tumor microenvironment. The main cellular component of tumor stroma is represented by an activated form of fibroblasts called Cancer-Associated Fibroblasts (CAFs). CAFs are widely recognized as key players in cancer progression [1,22] mainly by sustaining tumor cells survival and proliferation.

Typically, tumor tissue is both more acidic and relatively hypoxic compared to the normal counterpart. These peculiarities are commonly attributed to the defective vascularization of solid tumors that leads to an hypoxic environment that contributes to a metabolic shift of cancer cells metabolism towards glycolysis, with the consequent release of lactic acid in the extracellular milieu [11]. In addition HIF-induced CA IX in hypoxic cancer cells, together with bicarbonate transporters, generates a CO_2_/bicarbonate flux that serves to maintain the pHi permissive for cells survival through the intracellular import of bicarbonate and the extrusion of CO_2_[23]. The concept of pHe acidification mediated by cancer cells lactic acid release has been recently challenged by Fiaschi et al [8] that showed that in *in vitro* coculture glycolytic fibroblasts are, indeed, responsible for lactic acid production that is quantitatively imported by OXFOS cancer cells by means of MCTs transporters.

In the present work we studied the effect of pHe acidification and the effect of CA IX-mediated CO_2_/bicarbonate fluxes in an *in vitro* coculture model of prostatic tumor. Single culture of HPFs produced less pHe acidification compared to cancer cells (PC3 or DU145) both at 1 and 20% O_2_ (Figure 1) according to the common notion that highly proliferating cells, intensifying the glycolytic flux, lead to a strong production and release of acidic equivalents to control their pHi. Surprisingly, PC3-CAFs coculture, as well as DU145-CAFs one, showed a synergistic increment of acidic equivalents production. The proliferation index determined for each population in single and coculture system suggests: 1) PC3 or DU145 cells proliferation rate in single culture are highly sensitive to environmental pH. Acidic pHe is a stressful condition for tumor cells that leads to a notable reduction of proliferation (Figure 2A and B); 2) tumor cells, when cocultured with CAFs, show a significant increase in cell proliferation rate at every pHe considered. Nevertheless, the presence of CAFs is not sufficient to completely overcome the negative effect of acidic pHe (Figure 2A and B); 3) the transdifferentiation of HPFs to CAFs, induced by their coculture with tumor cells, leads to an increase in fibroblasts proliferation rate (Figure 2C), although at a lesser extent compared to that observed for cancer cells (Figure 2A and B); 4) hypoxic conditions, commonly found in tumor microenvironment, further contribute to enhance tumor cells proliferation rate both in single culture and in coculture (Figure 3).

In conclusion, coculture gives a proliferative advantage mainly to cancer cells, particularly in hypoxic condition, whereas pHe acidification constitutes a negative environmental condition. One of the proteins responsible for pHe acidification in the context of tumor microenvironment is CA IX [15,24], whose catalytic activity leads to extracellular production of protons and bicarbonate. Given that low pHe has a negative impact on both PC3 and DU145 proliferation, we have investigated the role of the other product of CO_2_ hydration, bicarbonate, in cancer cells proliferation. Summary data reported in Table 1 show that CAFs mediated DU145 cells proliferation rate enhancement relies almost totally on bicarbonate presence in the extracellular medium (Figure 4), while only 30% of PC3 cells proliferation increase due to the presence of CAFs is warranted by bicarbonate (Figure 5). A key question is why bicarbonate does not influence cells proliferation rate in single DU145 and PC3 culture (Figures 4A and 5A). In our opinion bicarbonate concentration sufficient for sustaining single culture cell proliferation can be obtained by atmospheric CO_2_ or by cell respiration derived CO_2_. On the contrary, when the growth rate of cancer cells is strongly increased, consequently to CAFs-mediated activation, bicarbonate concentration is the rate limiting ion both for DU145 and PC3 growth. Accordingly, in order to fulfill the demand of bicarbonate there is a strong upregulation of stromal CA IX when CAFs are cocultured with cancer cells (Figure 7). It has been shown that bicarbonate may serve as a shuttle to transport protons outside the cell. In this hypothesis, CO_2_ hydration by extracellular CAs produces protons and bicarbonate, then the latter is transported within the cell by bicarbonate transporters contributing to cytoplasm alkalinization [25,26] and reforming CO_2_. Our results, indeed, show that tumor cells also use bicarbonate as a mono carbonic fragment to build biosynthesis intermediates necessary for sustain their high proliferation rate (Figure 6 and Table 2) and that this process is enhanced in tumor cells cocultured with activated fibroblasts compared to cancer cells alone as indicated by the increased steady state concentrations of the two Krebs cycle intermediates analysed (Table 2).

The important role of stromal CA IX for sustaining cancer cells growth has been confirmed by siRNA mediated CA IX silencing in CAFs that leads to a remarkable decrease of DU145 proliferation respect to standard coculture conditions (Figure 8). The results of *in vitro* coculture model are also clearly confirmed by *in vivo* experiments in which we show that CAFs impaired in CA IX expression lead to a strong reduction of tumor growth (Figure 9).

## Conclusions

The possibility to counteract tumor progression depends on our understanding of cancer tissue physiology intended as a whole and not simply focusing on cancer cells behavior outside their physiological context. In the present work we show a new effect of functional cooperation established between CAFs and tumor cells in the context of the tumor microenvironment mediated by CA IX catalytic activity. In fact, the upregulation of stromal CA IX induced by cancer cells leads both to the acidification of extracellular milieu and to the production of bicarbonate. While extracellular acidification is disadvantageous for cell proliferation, bicarbonate, through its organication, supplies cancer cells with intermediates useful to sustain their high proliferation rate.

It is conceivable that in solid tumors the extracellular acidification could be maintained within acceptable limits by the dynamics of the extracellular fluid, while CA IX activity ensures the amount of bicarbonate sufficient for not constitute a limit to cancer cells growth rate.

## Methods

### Cell cultures

Human prostatic cancer cells (PC3 and DU145) were purchased from European Collection of Cell Culture. Healthy human prostatic fibroblasts (HPFs) were isolated from surgical explantation of patients who signed informed consent in accordance with the Ethics Committee of Azienda Ospedaliera Universitaria Careggi by Prof. Serni of Dipartimento di Medicina Sperimentale e Clinica/urology. Tumor cells and fibroblasts were routinely cultured in Dulbecco’s modified Eagle’s medium (DMEM) supplemented with 10% fetal bovine serum (Euroclone), 2 mM glutamine and penicillin (100 U/mL) and streptomycin (100 μg/mL) in a 5% CO_2_ humidified atmosphere at 37°C.

### Reagents

Unless otherwise specified all reagents are from Sigma/Aldrich. CA IX siRNA and all antibodies were purchased by Santa Cruz Biotechnologies, except the anti-CA IX antibodies that were from Bioscience Slovakia. Carboxyfluorescein diacetate succinimidyl ester (CFDA-SE), Opti-MEM and Lipofectamine are from Invitrogen. PVDF membrane and Immobilon Western Chemiluminescent HRP Substrate are from Millipore. Cocultures separation was performed by MACS MicroBeads and MACS Column Technology patented by Miltenyi Biotec. Apoptosis test is performed by Annexin-V-Fluos staining kit from Roche. Sodium [^14^C] bicarbonate was purchased from Perkin Elmer.

### Proliferation assay

The proliferation was evaluated using CFDA-SE. Tumor or stromal cells were labeled with the dye at the concentration of 2.5 μM. Then PC3, DU145 or fibroblasts were cultured alone or in coculture for 40 hours. After that the cells were detached, fixed in 3% paraformaldehyde and analyzed by flow cytometry. The fluorescence value obtained was analyzed by ModFit software to estimate the proliferation index. Proliferation assay was performed by growing cells in DME without sodium bicarbonate supplemented either with 25 mM hepes or with 25 mM hepes and 10 mM sodium bicarbonate. In the first the cells were grown in CO_2_/bicarbonate free environment, in the latter at 5% CO_2_. The media of each condition were brought at pH 6.8, pH 7.4 and pH 8.0.

### Small interfering RNA (siRNA) transfection

siRNA transfection was performed with Lipofectamine and Opti-MEM to knock down CA IX gene expression. CA IX siRNA is a pool of three target-specific 19–25 nt siRNAs; a scrambled siRNA was used as control. The cells were used for the experiments the day after the transfection.

### Acidification assay

The pH of the external medium of tumor cells or fibroblasts either in single cultures (2×10^5^ cells) or in cocultures (10^5^ fibroblasts and 10^5^ DU145 or PC3) was determined after 24 hours in DME supplemented with 1 mM sodium bicarbonate (pH 7.6) in atmospheric CO_2_.

### Western blot analysis

Cells were lysated with 2X SDS-Laemmli sample buffer without β-mercaptoethanol and bromophenol blue and protein concentration was evaluated by bicinchoninic acid (BCA) assay. After that, samples were precipitated in methanol/chloroform according to the following protocol: for about 150–300 μg of proteins contained in 150 μL of sample were added 600 μL of methanol, 150 μL of chloroform and 450 μL of water, after each addition the sample was mixed by vortexing. Then the sample was centrifuged at 13,500 g for 10 minutes, discarded the upper phase, kept the white precipitation disc evident between the upper and lower phases, and then 450 μL of methanol was added to the sample. After 10 minutes of centrifugation at 13,500 g the supernatant was discarded and the pellet was dried in a vacuum centrifuge for 20 minutes. Then, the sample was resuspended in SDS-Laemmli sample buffer. The proteins (40 μg for each sample) were separated by SDS-PAGE, transferred on PVDF membrane by Western Blot and probed with anti-CA IX antibody and with anti-actin antiboby as loading control. Immunoblots were incubated in phosphate buffered saline supplemented with 5% bovine serum albumin and 0.05% Tween for 1 hour at room temperature with primary antibody and then in phosphate buffered saline supplemented with 1% bovine serum albumin and 0.05% Tween for 45 minutes with secondary antibody conjugated with horseradish peroxidase (HRP). Development was performed with Immobilon Western Chemiluminescent HRP Substrate and chemiluminescence was detected by UVP (Ultra-Violet Products) Ltd Chemidoc-it 500 Imaging System. Quantitative analysis of the spots was carried out by Kodak MI software.

### Cocultures separation

DU145 or PC3 were labeled with CFDA-SE and then plated in coculture with fibroblasts in a 1:2 ratio. After 40 hours cells were detached with Accutase and separated with MACS Column Technology. A fraction of each cell sample was used to determine the separation efficiency by flow cytometry and the other part of the sample was lysated with 2X SDS-Laemmli sample buffer without β-mercapoethanol and bromophenol blue.

### Cell death determination

10^5^ DU145 or PC3 were plated in DME supplemented with 25 mM hepes and 10 mM sodium bicarbonate at pH 6.8, pH 7.4 and pH 8.0 for 40 hours at 5% CO_2_. Cells were detached with Accutase and labeled with Annexin-V-Fluos staining kit. The probe was directly detected by flow cytometry analysis.

### Sodium [^14^C] Bicarbonate incorporation

DU145 were plated in DME supplemented with 25 mM hepes, 10 mM sodium bicarbonate, 20 μM sodium [^14^C] bicarbonate (50.8 mCi/mmol) and 10% fetal bovine serum at pH 8.0 for 24, 48 and 72 hours. The medium was changed every day. The cells were lysed in 2X SDS-Laemmli sample buffer without β-mercapoethanol and bromophenol blue. Protein concentration was evaluated by bicinchoninic acid (BCA) assay. CPM (counts per minute) values were obtained by scintillation counter.

### GC-MS measurements

DU145 were plated in single culture and in coculture with fibroblasts in a 1:2 ratio in DME supplemented with 25 mM hepes and 25 mM sodium carbonate-^13^C (pH 8.0) at 5% CO_2_. The medium was changed every day. After 40 hours DU145 cells of single culture and DU145 cells separated from fibroblasts with MACS Column Technology were lysed in 100% cold (-80°C) ethanol. Ethanol soluble fraction was dried in a vacuum centrifuge. Dried cell extracts were redissolved in 100 μL of ethanol containing 1% acetic acid. The solution was transferred in a microvial, taken to dryness under a gentle stream of nitrogen at room temperature and then derivatized with N-methyl-N-(*t*-butyldimethylsilyl)trifluoroacetamide in the presence of 1% *t*-butyldimethylchlorosilane for 2 hours at 80°C. The samples were analyzed on an Agilent GC-MS system composed by a 7820A gas chromatograph coupled to a 5975B mass spectrometer operating in electron ionization (EI, 70 eV) mode (Agilent Technologies Italy, Cernusco sul Naviglio, Milan, Italy). The system was equipped with an automatic liquid sampler. The column was a DB 5 MS, 20 m, 0.18 mm, 0.18 μm film thickness (J&W, Agilent Technologies); helium was the carrier gas, at 0.7 ml/min constant flow rate. The oven temperature program was as follow: from 150°C to 220°C then at 5°C/min, then at 30°C/min to 310°C, maintained for 3 min. The injections were performed in split mode (4:1 split ratio); the injection volume was 1.5 μL. The injector port and transfer line temperatures were 260°C and 280°C, respectively. The ion source operated at 230°C, while the quadrupole temperature was 150°C. Data were acquired in SIM mode and alternating SIM/scan mode. In SIM the fragment ion at m/z (M - 57) was recorded for the ^12^C and mono ^13^C species in a specific retention time window: for scan acquisition mass spectra were acquired in the range from 40 m/z to 680 m/z. Data were acquired and elaborated using the MSD ChemStation software (version E.01.01.335, Agilent Technologies). The peaks for malate and fumarate from GC–MS signals were integrated, and atom percent excess (^13^C) values were calculated by comparison with control samples and unlabeled standard solutions. It was not possible to measure the percent enrichment in oxaloacetate because of the low concentration as well as the instability of this substance.

### Xenograft experiments

*In vivo* experiments were performed co-injecting PC3 and fibroblasts knocked down for CA IX gene or non-silenced fibroblasts in 6 to 8 week old male severe combined immunodeficient (SCID)-bg/bg mice (Charles River Laboratories International). Six animals were used *per* group, which were daily monitored. The tumor size was measured every two or three days by a caliper and the tumor volumes were determined by measuring the length (L) and the width (W) of the tumor and calculating the volume (V = LW^2^/2). Animals experiments were conducted in accordance with national guidelines and approved by the ethical committee of Animal Welfare Office of Italian Work Ministry and conform to the legal mandates and Italian guidelines for the care and maintenance of laboratory animals.

## Abbreviations

HPFs: Human primary fibroblasts; CAFs: Cancer associated fibroblasts; CA: Carbonic anhydrase; pHe: Extracellular pH; pHi: Intracellular pH; ECM: Extracellular matrix.

## Competing interests

The authors declare that they have no competing interests.

## Authors’ contributions

AS and AC performed most of the experiments, and analyzed the data. PP, DC, and MLT participated in experiments. GP performed mass spectrometry experiments and data analysis. SS, PCh and GC participated in the coordination of the study and critically revised the manuscript. PCi designed the research, analyzed the data, and wrote the article. All the authors read and approved the final manuscript.

## Supplementary Material

Additional file 1 **Analysis of CA IX expression in single culture and in coculture.** PC3 and fibroblasts were plated in single culture and in coculture in a 1:2 ratio for 40 hours at 1% O2. After that the coculture were detached and separated by MACS Column Technology. The samples were lysed in SDS-Laemmli Sample Buffer and used for Western Blot analysis. The membranes were treated with anti-CA IX and anti-actin antiboby. Lanes 1 and 2: HPFs and PC3 in single culture. Lanes 3 and 4: CAFs and PC3 after coculture and separation. The graph shows spots quantification using Kodak-MI software. The Western Blot is representative of four independent experiments with similar results. Click here for file
