# Supplementary material for: The effects of CA IX catalysis products within tumor microenvironment
Source: Cell Commun Signal. 2013 Oct 29;11:81. doi: 10.1186/1478-811X-11-81 (PMC3874735; doi:10.1186/1478-811X-11-81)
Supplement: Additional file 1 — Analysis of CA IX expression in single culture and in coculture. PC3 and fibroblasts were plated in single culture and in coculture in a 1:2 ratio for 40 hours at 1% O2. After that the coculture were detached and separated by MACS Column Technology. The samples were lysed in SDS-Laemmli Sample Buffer and used for Western Blot analysis. The membranes were treated with anti-CA IX and anti-actin antiboby. Lanes 1 and 2: HPFs and PC3 in single culture. Lanes 3 and 4: CAFs and PC3 after coculture and separation. The graph shows spots quantification using Kodak-MI software. The Western Blot is representative of four independent experiments with similar results. [file 1478-811X-11-81-S1.pdf]

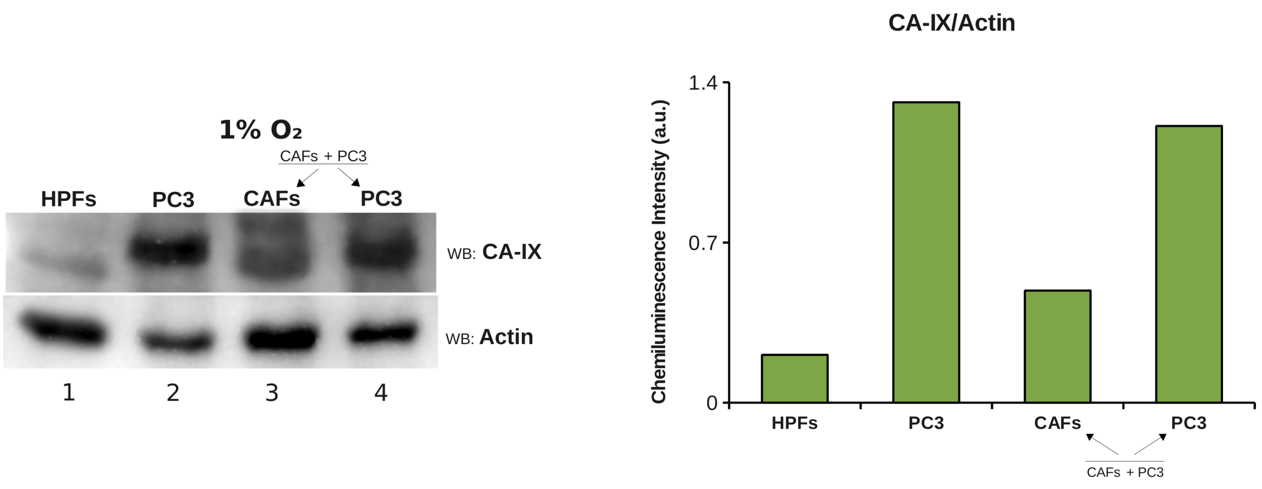

Additional file 1. Analysis of CA IX expression in single culture and in coculture. PC3 and fibroblasts were plated in single culture and in coculture in a 1:2 ratio for 40 hours at 1% O_2. After that the coculture were detached and separated by MACS Column Technology. The samples were lysed in SDS-Laemmli Sample Buffer and used for Western Blot analysis. The membranes were treated with anti-CA IX and anti-actin antiboby. Lanes 1 and 2: HPFs and PC3 in single culture. Lanes 3 and 4: CAFs and PC3 after coculture and separation. The graph shows spots quantification using Kodak-MI software. The Western Blot is representative of four independent experiments with similar results.
